# Association Between Laboratory Values and Covert Hepatic Encephalopathy in Patients with Liver Cirrhosis: A Multicenter, Retrospective Study

**DOI:** 10.3390/jcm14061858

**Published:** 2025-03-10

**Authors:** Kaori Koyano, Masanori Atsukawa, Akihito Tsubota, Chisa Kondo, Takao Miwa, Tadashi Namisaki, Atsushi Hiraoka, Hidenori Toyoda, Toshifumi Tada, Yuji Kobayashi, Kazuhito Kawata, Kentaro Matsuura, Shigeru Mikami, Naoto Kawabe, Tsunekazu Oikawa, Kenta Suzuki, Tadamichi Kawano, Tomomi Okubo, Taeang Arai, Joji Tani, Asahiro Morishita, Motoh Iwasa, Toru Ishikawa, Tadashi Ikegami, Yasuhito Tanaka, Masahito Shimizu, Hitoshi Yoshiji, Katsuhiko Iwakiri

**Affiliations:** 1 Division of Gastroenterology and Hepatology, Nippon Medical School, Tokyo 113-8602, Japan; s11-046sk@nms.ac.jp (K.K.); s8042@nms.ac.jp (C.K.); s11-052sk@nms.ac.jp (K.S.); k-tadamichi@nms.ac.jp (T.K.); taeangpark@yahoo.co.jp (T.A.); k-iwa@nms.ac.jp (K.I.); 2 Project Research Units, Research Center for Medical Science, The Jikei University School of Medicine, Tokyo 105-8461, Japan; atsubo@jikei.ac.jp (A.T.); oitsune@jikei.ac.jp (T.O.); 3 Department of Gastroenterology/Internal Medicine, Graduate School of Medicine, Gifu University, Gifu 501-1193, Japanshimizu.masahito.j1@f.gifu-u.ac.jp (M.S.); 4 Department of Gastroenterology, Nara Medical University, Nara 634-8521, Japan; tadashin@naramed-u.ac.jp (T.N.); yoshijih@naramed-u.ac.jp (H.Y.); 5 Gastroenterology Center, Ehime Prefectural Central Hospital, Matsuyama 790-0024, Japan; hirage@gmail.com; 6 Department of Gastroenterology, Ogaki Municipal Hospital, Gifu 503-8502, Japan; hmtoyoda@spice.ocn.ne.jp; 7 Department of Internal Medicine, Japanese Red Cross Himeji Hospital, Himeji 670-8540, Japan; tadat0627@gmail.com; 8 Department of Hepatology, Saiseikai Niigata Hospital, Niigata 950-1104, Japan; y.kobayashi@ngt.saiseikai.or.jp (Y.K.); toruishi@ngt.saiseikai.or.jp (T.I.); 9 Hepatology Division, Department of Internal Medicine II, Hamamatsu University School of Medicine, Hamamatsu 431-3125, Japan; kawata@hama-med.ac.jp; 10 Department of Gastroenterology and Metabolism, Nagoya City University Graduate School of Medical Sciences, Nagoya 464-0083, Japan; matsuura@med.nagoya-cu.ac.jp; 11 Department of Internal Medicine, Division of Gastroenterology, Kikkoman General Hospital, Noda 278-0005, Japan; smikami@mail.kikkoman.co.jp; 12 Department of Gastroenterology and Hepatology, School of Medicine, Fujita Health University, Toyoake 470-1192, Japan; kawabe@fujita-hu.ac.jp; 13 Department of Gastroenterology, Nippon Medical School Chiba Hokusoh Hospital, Inzai 270-1694, Japan; ma6-0154@nms.ac.jp; 14 Department of Gastroenterology, Kagawa University Graduate School of Medicine, Kagawa 761-0793, Japan; tani.joji@kagawa-u.ac.jp (J.T.); morishita.asahiro@kagawa-u.ac.jp (A.M.); 15 Department of Gastroenterology and Hepatology, Mie University School of Medicine, Tsu 514-8507, Japan; motoh@med.mie-u.ac.jp; 16 Division of Hepatology and Gastroenterology, Department of Internal Medicine, Tokyo Medical University Ibaraki Medical Center, Ibaraki 300-0332, Japan; ikegamit@tokyo-med.ac.jp; 17 Department of Gastroenterology and Hepatology, Faculty of Life Sciences, Kumamoto University, Kumamoto 860-8555, Japan; ytanaka@kumamoto-u.ac.jp

**Keywords:** covert hepatic encephalopathy, 25(OH)D3, liver cirrhosis, Stroop test

## Abstract

**Background/Objective**: Recently, there has been an increasing need to implement the diagnosis of the presence of covert hepatic encephalopathy (CHE) in patients with cirrhosis. The aim of this study was to identify novel factors associated with CHE in clinical practice. **Methods**: This retrospective study enrolled a total of 402 patients with cirrhosis at 17 institutions. The Stroop test was performed to diagnose CHE at each center. **Results**: The patients comprised 233 males and 169 females, with a median age of 69 (IQR, 61–75) years. The median albumin and 25(OH)D3 levels were 3.9 (3.5–4.3) g/dL and 15.4 (11.0–21.0) ng/mL, respectively. This cohort included 181 patients with esophageal varices (EV). Multivariate analysis revealed that low 25(OH)D3 (*p* < 0.05) and EV (*p* < 0.05) were independent risk factors for CHE. When limited to only laboratory factors, low albumin (*p* < 0.01) and low 25(OH)D3 (*p* < 0.05) were independent factors for CHE. The optimal cut-off values of albumin and 25(OH)D3 for predicting CHE were 3.7 g/dL and 16.5 ng/mL, respectively. The prevalence of CHE was 59.2% for 25(OH)D3 < 16.5 ng/mL and EV, 53.8% for albumin < 3.7 g/dL and 25(OH)D3 < 16.5 ng/mL, and 66.7% for albumin < 3.7 g/dL, EV, and 25(OH)D3 < 16.5 ng/mL. **Conclusions**: Low 25(OH)D3 and albumin levels, and the EV were positively associated with CHE in patients with cirrhosis. Specifically, the prevalence of CHE increased with a decrease in 25(OH)D3 levels. Patients with such risk factors should be actively and carefully examined for the presence of CHE.

## 1. Introduction

Hepatic encephalopathy is one of the most serious complications of cirrhosis. Patients with hepatic encephalopathy present with nonspecific neuropsychiatric symptoms, ranging from imperceptible impairment in mental status to deep coma. Hepatic encephalopathy is classified into two categories: covert hepatic encephalopathy (CHE; manifesting minimal or mild clinical symptoms) and overt hepatic encephalopathy (OHE; manifesting obvious clinical symptoms) [1].

Approximately 20–60% of patients with cirrhosis have CHE, and its frequency increases with worsened hepatic functional reserve [2,3,4]. The routine treatment modalities for OHE include diet therapy [such as branched-chain amino acid (BCAA) and late evening snack] and medications (such as nonabsorbable disaccharides, nonabsorbable antimicrobial agents, carnitine preparations, zinc preparations, and probiotics). Balloon-occluded retrograde transvenous obliteration has been reported to be effective for shunt hepatic encephalopathy [1,5]. Although these treatments are indicated for OHE, there is no clear consensus on the need for therapeutic intervention in patients with CHE.

CHE is causally related to reduced quality of life, increased frequency of traffic accidents, increased sleep disturbances, increased risk of falls, and decreased work capacity [6,7,8,9,10]. Furthermore, minimal hepatic encephalopathy, defined as a part of CHE in the West Haven Criteria, is also associated with mortality in patients with cirrhosis independent of the complications of hepatocellular carcinoma and the severity of liver damage [2]. Recently, the European Association for the Study of the Liver (EASL) practice guidelines for hepatic encephalopathy recommend treatment of CHE with drugs, such as nonabsorbable disaccharides and rifaximin, owing to the high risk of transition to OHE [11]. Thus, there is a growing recognition of the importance of diagnosing CHE in patients with cirrhosis. While patients with CHE retain verbal cognitive abilities, they have a noticeable decline in motor cognitive abilities. Neuropsychiatric function tests (NPTs), such as the number connection test, block design test, and digital symbol test, have been known to be useful for diagnosing hepatic encephalopathy. Additionally, the usefulness of the Stroop test has been recently reported [5,12,13]. Compared to conventional NPTs, the Stroop test is easier to perform, and the examination time is reduced to approximately 5–10 min [13]. Nevertheless, it is difficult to devote sufficient time and personnel to perform the Stroop test on all patients with cirrhosis in an outpatient setting.

Alternatively, several simple markers that could predict CHE have been reported. In addition to serum albumin [13,14] and zinc levels [15], the severity of hepatic damage, blood ammonia levels, and esophageal varices have been reported to be useful in predicting CHE [3,13,14]. Although the classical active form of vitamin D plays a major role in increasing the serum calcium concentration by promoting absorption of calcium from the intestinal tract or increasing bone resorption [16], serum 25(OH)D3 levels decrease with reduced hepatic functional reserve [17]. Reportedly, low 25(OH)D3 levels were associated with the development of OHE [17,18,19,20,21]. In addition, low serum 25(OH)D3 levels are closely associated with low skeletal muscle mass and sarcopenia [17,21]. In patients with cirrhosis, the skeletal muscle compensates for impaired hepatic ammonia detoxification capacity by using BCAA as a substrate. These findings may indicate an indirect, rather than direct, cross-relationship between serum 25(OH)D3 levels and CHE. However, to our knowledge, there are no reports on the association between serum 25(OH)D3 levels and CHE.

Therefore, this multicenter study aimed to identify factors positively associated with CHE and to identify novel predictive simple markers that are readily available in clinical practice.

## 2. Materials and Methods

### 2.1. Patients

This was a retrospective study of 553 patients with liver cirrhosis who were diagnosed with CHE using the Stroop test at 17 institutions between November 2023 and April 2024. The inclusion criteria were as follows: (1) diagnosis of cirrhosis and (2) patient age ≥ 20 years. The exclusion criteria were as follows: (1) untreatable hepatocellular carcinoma (HCC); (2) history of OHE, e.g., those who have been treated with anti-hepatic encephalopathy drugs; (3) history of brain injury or stroke; (4) history of neurological/psychiatric disease; (5) color blindness; and (6) vitamin D supplementation before or at the entry. Cirrhosis was diagnosed through imaging (abdominal computed tomography and/or ultrasonography) or liver biopsy. Child–Pugh classification and ALBI grade were used to assess liver functional reserve. Albumin–Bilirubin (ALBI) grade was calculated based on serum albumin and total bilirubin values using the following formula: ALBI score = [log10 bilirubin (µmol/L) × 0.66] + [albumin (g/L) × −0.085]. Grip strength was measured twice using a Smedley-type grip force meter for each of the right and left hands, and the average of the higher right- and left-sided values was recorded as the grip strength value. Bioelectrical impedance analysis was performed using InBody 270 (Biospace, Seoul, Republic of Korea) to estimate appendicular skeletal muscle mass, which was calculated as the sum of lean muscle masses of the bilateral upper and lower extremities. The skeletal muscle mass index (SMI) was calculated as follows: SMI (kg/m^2^) = appendicular skeletal muscle mass (kg)/[height (m)]^2^. Baseline hematological, biochemical, and clotting test results were collected from data records. Serum 25-hydroxyvitamin D [25(OH)D3] levels that are representative of serum vitamin D levels were measured using a double-antibody radioimmunoassay kit (SRL, Tokyo, Japan).

### 2.2. Stroop Test

The Stroop test was performed to diagnose CHE at each center according to previous reports [22,23,24]. Age-specific cutoff values for CHE diagnosis determined in a previous study of Japanese subjects were applied [13]. The Stroop test software (Otsuka Pharmaceutical Co., Ltd., Tokyo, Japan) was distributed by the Japan Society of Hepatology and was installed on the iPad (Apple Computer, Cupertino, CA, USA).

### 2.3. Ethical Statement

The present study was conducted following the ethical guidelines established in the 2013 Helsinki Declaration and was approved by the ethics committee of Nippon Medical School (approval no. M-2022-067, approval date 12 July 2023). Patients were given the option to abstain from participating in this study.

### 2.4. Statistical Analyses

Categorical data are expressed as numbers, while continuous data are expressed as medians and interquartile ranges (IQRs). Fisher’s exact test and Mann–Whitney U test were used to compare two groups, as appropriate. Univariate and multiple logistic regression analyses were performed to identify significant independent risk factors for CHE. Receiver operating characteristic (ROC) curve analysis was performed to determine the optimal cut-off values of serum 25(OH)D3 and albumin to predict CHE. Sensitivity and specificity were calculated for the determined optimal cut-off values or the presence or absence of esophageal varices. The Cochran–Armitage trend test was used to evaluate a significant trend toward an increase in the prevalence of CHE with a decrease in serum 25(OH)D3 levels. All statistical analyses were performed using SPSS version 26.0 (IBM Corporation, Armonk, NY, USA). Differences with *p* < 0.05 were considered statistically significant.

## 3. Results

### 3.1. Patient Characteristics

Of the 553 enrolled patients with liver cirrhosis, 151 were excluded (missing data = 123, history of OHE = 28); thus, 402 patients were eligible for this study analysis (Supplementary Figure S1). This cohort comprised 233 males and 169 females, with a median age of 69 (IQR, 61–75) years. The numbers of patients with Child–Pugh class A, B, and C were 271, 102, and 29, respectively. The median ALBI score was −2.56 (−2.90–−2.13). The median values for platelet count, albumin, total bilirubin, prothrombin time, and blood ammonia were 127 (93.0–177.0) × 10^3^/μL, 3.9 (3.5–4.3) g/dL, 1.0 (0.7–1.5) mg/dL, 87.0 (70.0–100.0)%, and 44.0 (30.0–66.0) µg/dL, respectively. The median FIB-4 index was 3.37 (2.21–5.28). The median serum 25(OH)D3 and zinc levels were 15.4 (11.0–21.0) ng/mL and 68 (59–81) µg/dL, respectively. This cohort included 181 patients with esophageal varices. Other clinical characteristics at baseline are summarized in Table 1.

### 3.2. Comparison of Baseline Characteristics Between Patients with and Without Covert Hepatic Encephalopathy

CHE was present in 146 (36.3%) of the 402 patients. Table 1 shows the baseline characteristics of patients with and without CHE. Total bilirubin (*p* < 0.05), blood ammonia (*p* < 0.01), ALBI score (*p* < 0.001), and FIB-4 index (*p* < 0.01) in patients with CHE were significantly higher than those in patients without CHE. Conversely, platelet count (*p* < 0.05), albumin (*p* < 0.01), prothrombin time (*p* < 0.05), 25(OH)D3 (*p* < 0.01), and zinc (*p* < 0.05) in patients with CHE were significantly lower than those in patients without CHE. The prevalence of esophageal varices was significantly higher in patients with CHE than in those without CHE (*p* < 0.001).

### 3.3. Factors Associated with Covert Hepatic Encephalopathy

Among the 402 patients, univariate and multivariate regression analyses identified serum 25(OH)D3 (odds ratio [OR], 1.037; 95% confidence interval [CI], 1.005–1.069; *p* < 0.05) and esophageal varices (OR, 1.622; 95% CI, 1.042–2.525; *p* < 0.05) as significant factors associated with CHE (Table 2). In clinical practice, not all patients with cirrhosis undergo esophagogastroduodenoscopy. Therefore, the present study re-analyzed data using laboratory factors alone (excluding invasive examination). When limited to only laboratory factors, low serum albumin (OR, 1.047; 95% CI, 1.011–1.085; *p* < 0.01) and low serum 25(OH)D3 (OR, 1.032; 95% CI, 1.002–1.063; *p* < 0.05) were positively associated with CHE (Table 3).

Considering that it may be difficult to distinguish CHE from geriatric mental disorder (including dementia) in patients aged 75 years and over, we re-analyzed the factors associated with CHE only in 286 patients under 75 years. Univariate and multivariate regression analyses identified serum 25(OH)D3 (OR, 1.057; 95% CI, 1.018–1.098; *p* < 0.01) and esophageal varices (OR, 2.123; 95% CI, 1.286–3.505; *p* < 0.01) as significant factors associated with CHE (Supplementary Table S1). When limited to only laboratory factors, low serum albumin (OR, 1.066; 95% CI, 1.024–1.111; *p* < 0.01) and low serum 25(OH)D3 (OR, 1.039; 95% CI, 1.003–1.076; *p* < 0.05) were positively associated with CHE (Supplementary Table S2).

### 3.4. Optimal Cut-Off Values for Predicting Covert Hepatic Encephalopathy

**Figure 1:**
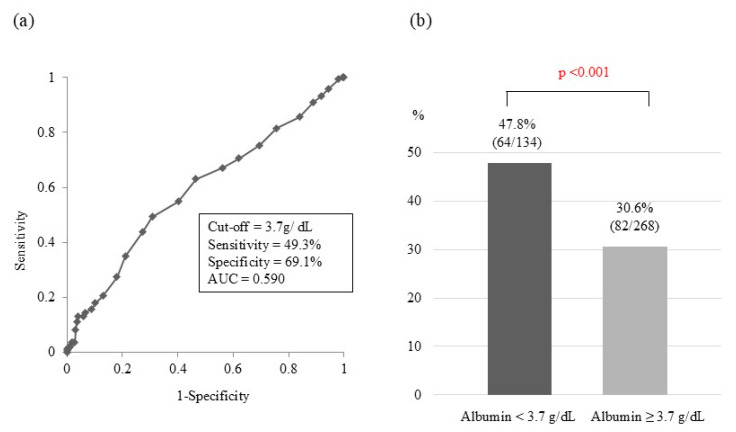
Receiver operator characteristic curve analysis of the predictive value of serum albumin for covering hepatic encephalopathy. (**a**) The optimal serum albumin cut-off value for predicting cover hepatic encephalopathy was 3.7 g/dL. AUC, area under the curve. (**b**) Prevalence of cover hepatic encephalopathy according to the cut-off serum albumin value.

**Figure 2:**
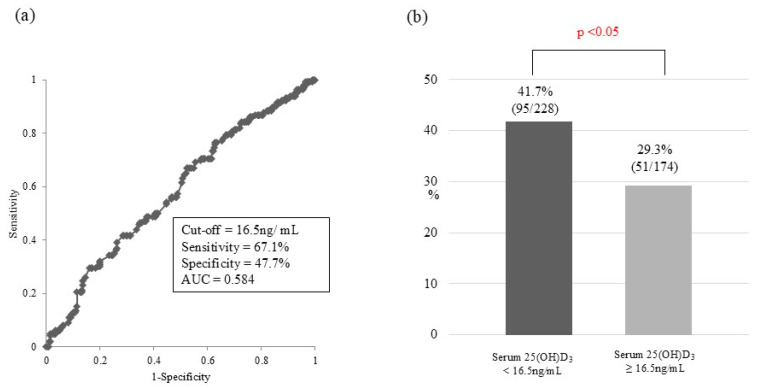
Receiver operator characteristic curve analysis of the predictive value of serum 25(OH)D3 for covering hepatic encephalopathy. (**a**) The optimal cut-off serum 25(OH)D3 level value for predicting cover hepatic encephalopathy was 16.5 ng/mL. AUC, area under the curve. (**b**) Prevalence of cover hepatic encephalopathy according to the cut-off serum 25(OH)D3 value.

**Figure 3:**
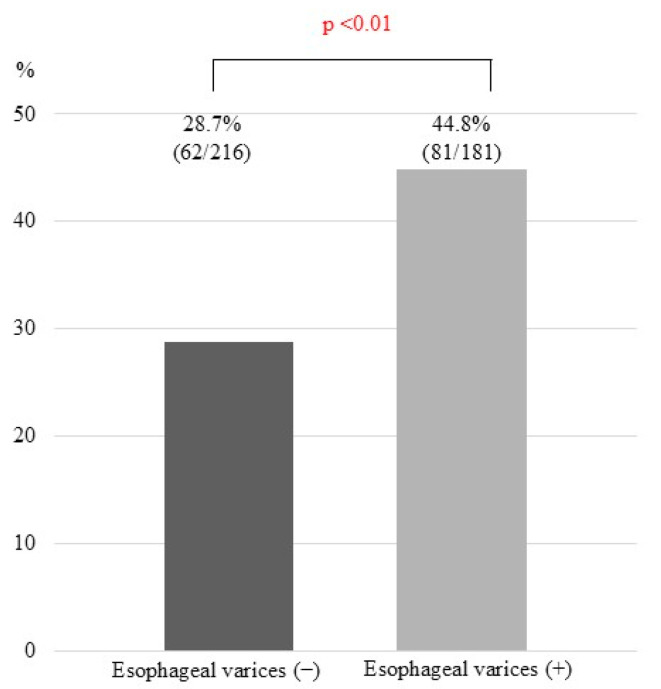
Prevalence of patients with covered hepatic encephalopathy with or without esophageal varices.

In ROC analysis, the optimal cut-off value of serum albumin for predicting CHE in the entire cohort (*n* = 402) was 3.7 g/dL [area under the curve (AUC), 0.590; sensitivity, 49.3%; specificity, 69.1%; Figure 1a]. When the patients were divided into two groups using this cut-off value, the prevalence of CHE in patients with serum albumin < 3.7 g/dL was significantly higher than that in patients with serum albumin ≥ 3.7 g/dL [47.8% vs. 30.6%; *p* < 0.001; Figure 1b]. Regarding serum 25(OH)D3, the optimal cut-off value for predicting CHE was 16.5 ng/mL (AUC, 0.584; sensitivity, 67.1%; specificity, 47.7%; Figure 2a). Using this cut-off value, the prevalence of CHE in patients with serum 25(OH)D3 < 16.5 ng/mL was significantly higher than that in patients with serum 25(OH)D3 ≥ 16.5 ng/mL [41.7% vs. 29.3%; *p* < 0.05; Figure 2b]. Concerning esophageal varices, the prevalence of CHE was significantly higher in patients with them than in those without them (44.8 vs. 28.7%; *p* < 0.01; Figure 3). The sensitivity and specificity of esophageal varices for predicting CHE were 71.3% and 44.8%, respectively.

When limited to 286 patients under 75 years, the optimal cut-off value of serum albumin for predicting CHE was 3.7 g/dL [AUC, 0.628; sensitivity, 49.1%; specificity, 73.8%; Supplementary Figure S2a]. When the patients were divided into two groups using this cut-off value, the prevalence of CHE in patients with serum albumin < 3.7 g/dL was significantly higher than that in patients with serum albumin ≥ 3.7 g/dL [55.6% vs. 32.7%; *p* < 0.001; Supplementary Figure S2b]. Regarding serum 25(OH)D3, the optimal cut-off value for predicting CHE was 16.4 ng/mL [AUC, 0.613; sensitivity, 71.1%; specificity, 47.7%; Supplementary Figure S3a]. Using this cut-off value, the prevalence of CHE in patients with serum 25(OH)D3 < 16.4 ng/mL was significantly higher than that in patients with serum 25(OH)D3 ≥ 16.4 ng/mL [47.3% vs. 29.1%; *p* < 0.01; Supplementary Figure S3b]. Concerning esophageal varices, the prevalence of CHE in patients with them was significantly higher than that in those without them (50.8 vs. 10.2%; *p* < 0.001; Supplementary Figure S4). The sensitivity and specificity of esophageal varices for predicting CHE were 69.9% and 50.8%, respectively. These findings suggest that the predictive performance of laboratory factors for CHE improves after excluding elderly patients who may have subclinical geriatric psychiatric disability.

### 3.5. Prevalence of Covert Hepatic Encephalopathy According to Combined Risk Factors

**Figure 4:**
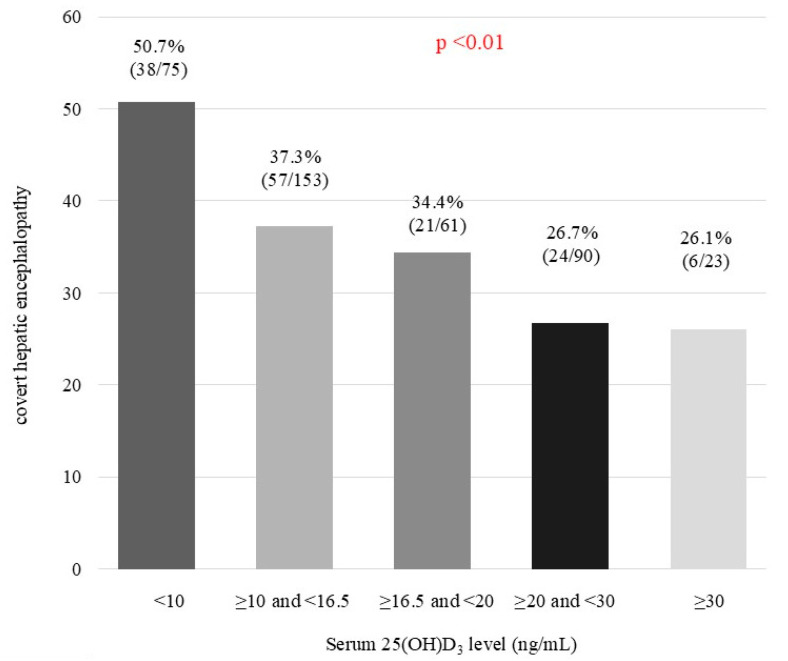
Prevalence of cover hepatic encephalopathy according to serum 25(OH)D3 levels. Statistical analysis was performed using the Cochran–Armitage trend test.

Supplementary Figure S5 shows the prevalence of CHE according to the combination of risk factors. The prevalence of CHE was 50.0% (47 of 94) for patients with serum albumin < 3.7 g/dL and esophageal varices, 52.2% (59 of 113) for those with serum 25(OH)D3 < 16.5 ng/mL and esophageal varices, 53.8% (50 of 93) for those with serum albumin < 3.7 g/dL and serum 25(OH)D3 < 16.5 ng/mL, and 66.7% (40 of 66) for those with all three risk factors. When the combined risk factors included serum 25(OH)D3, the prevalence of CHE was relatively high. Therefore, we focused on the prevalence of CHE according to serum 25(OH)D3 levels: 50.7% (38 of 75), 37.3% (57 of 153), 34.4% (21 of 61), 26.7% (24 of 90), and 26.1% (6 of 23) for patients with 25(OH)D3 < 10 ng/mL, ≥10 ng/mL and <16.5 ng/mL, ≥16.5 ng/mL and <20 ng/mL, ≥20 ng/mL and <30 ng/mL, and ≥30 ng/mL (*p* < 0.01; Figure 4).

## 4. Discussion

This study identified several factors that could be simply predictive of CHE and characterized patients with cirrhosis who should be more aggressively examined for the diagnosis of CHE. Low serum albumin levels and the presence of esophageal varices have been previously reported as significant risk factors for CHE [4,13]. Our findings are consistent with previous studies. However, no studies have identified low serum 25(OH)D3 levels as a factor associated with CHE. Adding serum 25(OH)D3 levels as a novel factor may help identify patients who should be evaluated for CHE. Upper gastrointestinal endoscopy should often be performed in patients with cirrhosis, especially in those with decompensated cirrhosis where hepatic encephalopathy is suspected, and not as an additional test for the diagnosis of CHE. This study also highlights that serum 25(OH)D3 and albumin levels, which are predictors of CHE, are important and readily available factors in the management of patients with cirrhosis, without the factor of the presence of esophageal varices diagnosed by upper gastrointestinal endoscopy.

Albumin is synthesized in the liver; therefore, deteriorated hepatic functional reserve causes a decrease in serum albumin levels. It has been reported that depleted hepatic functional reserve is closely associated with an increased risk of CHE. In a study of 117 patients with cirrhosis in Germany, high MELD scores and low serum albumin levels showed an independent association with CHE [3]. In the present study, patients with CHE had a significantly lower hepatic functional reserve than patients without CHE. The cut-off values of serum albumin levels for predicting CHE were 3.2 g/dL in one study of 311 patients with cirrhosis [13] and 3.15 g/dL in another study of 27 patients with cirrhosis aged under 65 years and without portosystemic shunt [14]. In the present study, the calculated serum albumin values (3.7 g/dL) were higher than the best cut-off value of 3.15 g/dL for albumin levels predictive of CHE reported by Kaji et al. [14]. The report by Kaji et al. was based on a cohort from a clinical trial of rifaximin for hepatic encephalopathy, which may have excluded patients with a history of HCC. This study included 101 patients with HCC, which may suggest that patients with higher albumin levels may also be at risk of CHE in a cohort closer to the real clinical setting. In addition, Kaji et al. [14] used the NP test to diagnose CHE, which is different from the Stroop test in the present study. On the other hand, validation in a larger number of patients in different cohorts may be needed to determine the best cut-off values for serum albumin levels to predict CHE.

Low serum 25(OH)D3 levels are closely associated with low skeletal muscle mass and sarcopenia, and vice versa [17,21]. However, the present study failed to show an association between skeletal muscle mass and CHE. As described above, low serum albumin levels were significantly associated with CHE. In our previous study of 619 patients with chronic hepatitis C, low serum albumin levels were found to be associated with vitamin D deficiency [serum 25(OH)D3 level < 20 ng/mL]. Therefore, serum 25(OH)D3 levels may be a surrogate marker for impaired hepatic functional reserve that could cause CHE. The EASL clinical practice guidelines recommend vitamin D supplementation for patients with cirrhosis who have vitamin D deficiency [25]. However, it is unclear whether vitamin D administration improves or prevents CHE.

The NPTs and Stroop test have limited ability to differentiate CHE from senile mental disturbance in the elderly. The diagnostic performance of the Stroop test was reported to be lower in patients aged over 75 years [13]. In a study by the U.S. Department of Veterans Affairs, 5647 (7.89%) of 71,552 veterans with cirrhosis had a diagnosis of dementia, indicating the need for increased awareness of the overlap between dementia and HE in elderly patients with cirrhosis [26]. Furthermore, Kaji et al. [14] reported that the first important factor in the diagnosis of CHE is age and indeed showed that the predictive power of each factor, such as serum albumin, increased when the cohort was restricted to those younger than 65 years. The present study also showed that the predictive ability of serum albumin level improved from an AUC of 0.590 to 0.628 in an analysis restricted to those aged <75 years. In addition, the predictive ability of serum 25(OH)D3 level also improved from an AUC of 0.584 to 0.613 in an analysis restricted to those aged <75 years. Therefore, the present study focused on patients under 75 years and suggested that the predictive performance for CHE improves by excluding elderly patients who may have latent psychiatric disorders. Elderly patients diagnosed with CHE should be examined for mental disability (including dementia) at the same time.

Some limitations of this study should be acknowledged. First, given that not all patients with cirrhosis undergo esophagogastroduodenoscopy in clinical practice, this study evaluated only the simple laboratory factors that are routinely measured. Although serum albumin and 25(OH)D3 levels were significant predictors of CHE, their predictive performances were suboptimal. Routinely available laboratory examinations did not have high sensitivity and specificity for predicting CHE. Therefore, it is imperative to explore other convenient, noninvasive markers with high predictive performance for CHE. Second, selection bias may have occurred in this study as it was a retrospective cohort in which 151 (27%) were excluded due to missing data or history of OHE in case selection. Validation in future studies using cohorts with a large number of prospective cases and no missing data is needed. Third, five patients with primary biliary cholangitis were included in this study, and all had vitamin D levels below 20 ng/mL, but none had CHE. Vitamin D levels are predicted to be low in cholestatic liver diseases such as primary biliary cholangitis and therefore may not be useful in predicting CHE. Fourth, whether vitamin D supplementation improves CHE has not been examined and needs to be investigated in the future. Indeed, the pathophysiological role of vitamin D on CHE in patients with cirrhosis remains unclear and requires further research. Lastly, vitamin D is synthesized mainly in the skin as well as being ingested from food. With ultraviolet irradiation, 7-dehydrocholesterol in the skin is converted to pre-vitamin D3. Position 25 is hydroxylated in the liver and metabolized to 25(OH)D3 [27]. Although we gathered data on serum 25(OH)D3, data on the number of hours of sunshine per day were not available.

## 5. Conclusions

Low serum 25(OH)D3 levels, low serum albumin levels, and the presence of esophageal varices were positively associated with CHE in patients with cirrhosis, particularly in those aged <75 years. Specifically, the prevalence of CHE increased with a decrease in serum 25(OH)D3 levels. The use of combined risk factors, including serum 25(OH)D3 levels, improved the predictive performance for CHE. Patients with such risk factors should be actively and carefully examined for the presence of CHE.

## Figures and Tables

**Table 1:** Baseline characteristics and comparison of baseline characteristics between patients with and without covert hepatic encephalopathy.

Variable	N = 402	CHE (−), N = 256	CHE (+), N = 146	*p* Value
Gender (Male/Female)	233/169	146/110	87/59	0.617
Age (years)	69 (61–75)	71 (64–76)	65 (57–74)	<0.001
Platelet count (×10^3^/μL)	127.0 (93.0–177.0)	132.0 (96.8–185.0)	119.0 (80.3–158.8)	<0.05
AST (U/L)	31 (24–42)	32 (24–45)	29 (24–40)	0.278
ALT (U/L)	22 (16–32)	22 (16–33)	22 (15–30)	0.525
Serum albumin (g/dL)	3.9 (3.5–4.3)	4.0 (3.6–4.3)	3.8 (3.4–4.2)	<0.01
Total bilirubin (mg/dL)	1.0 (0.7–1.5)	1.0 (0.7–1.4)	1.1 (0.7–1.7)	<0.05
Prothrombin time (%)	87.0 (70.0–100.0)	89.4 (73.6–100.0)	82.2 (65.0–100.0)	<0.05
BUN (mg/dL)	16.0 (12.6–19.1)	16.0 (12.8–19.1)	15.6 (12.4–19.6)	0.786
Creatinine (mg/dL)	0.78 (0.65–0.95)	0.78 (0.64–0.97)	0.78 (0.65–0.93)	0.987
Sodium (Meq/L)	140 (138–142)	140 (138–142)	140 (138–142)	0.553
Blood ammonia (µg/dL)	44.0 (30.0–66.0)	43.0 (29.0–58.0)	48.6 (31.1–83.0)	<0.01
Serum 25(OH)D_3_ (ng/mL)	15.4 (11.0–21.0)	16.0 (11.5–21.9)	14.8 (9.9–18.4)	<0.01
Zinc (µg/dL)	68 (59–81)	71.0 (60.5–83.5)	66.0 (56.0–77.0)	<0.05
Child-Pugh class (A/B/C)	271/102/29	208/42/6	63/60/23	<0.001
ALBI score	−2.56 (−2.90–−2.13)	−2.60 (−2.91–−2.22)	−2.36 (−2.79–−1.98)	<0.001
ALBI grade (1/2a/2b/3)	186/88/100/28	129/60/55/12	57/28/45/16	<0.01
FIB-4 index	3.37 (2.21–5.28)	3.10 (1.96–5.01)	3.83 (2.47–5.88)	<0.01
Esophageal varices (absence/presence/unknown)	216/181/5	154/100/2	62/81/3	<0.001
SMI (kg)	15.6 (12.3–19.7)	15.6 (12.4–19.5)	16.0 (11.9–20.9)	<0.05
Grip strength	26.2 (20.00–34.5)	26.3 (20.5–34.1)	25.9 (19.2–35.5)	0.641

Data are presented as numbers or median (inter interquartile range) values. CHE, covert hepatic encephalopathy; AST, aspartate aminotransferase; ALT, alanine aminotransferase; FIB-4, fibrosis-4; SMI, skeletal muscle mass index.

**Table 2:** Factors associated with covert hepatic encephalopathy.

		Univariate			Multivariate		
Variable	Category	OR	95% CI	*p* Value	OR	95% CI	*p* Value
Gender	1: Female	0.900	0.596–1.360	0.221			
Age	by 1 year up	0.968	0.949–0.987	<0.001			
Platelet count	by 1.0 × 10^3^/μL down	1.003	1.000–1.006	0.198			
Total bilirubin	by 0.1 mg/dL up	1.037	1.013–1.062	<0.01			
Prothrombin time	by 1.0% down	1.011	1.001–1.021	0.027			
Serum albumin	by 0.1 g/dL down	1.056	1.021–1.093	<0.01			
BUN	by 1.0 mg/dL up	0.993	0.962–1.025	0.667			
Creatinine	by 0.1 mg/dL up	1.006	0.973–1.039	0.730			
Sodium	by 1.0 meq/L down	1.018	0.949–1.091	0.621			
Blood ammonia	by 10 µg/dL up	1.152	1.079–1.229	<0.0001			
Serum 25(OH)D_3_	by 1.0 ng/mL down	1.041	1.011–1.071	<0.01	1.037	1.005–1.069	<0.05
Zinc	by 1.0 µg/dL down	1.012	0.999–1.025	0.052			
Esophageal varices	presence	2.012	1.328–3.049	<0.001	1.622	1.042–2.525	<0.05
SMI	by 1.0 kg down	0.931	0.873–0.992	0.028			
grip strength	By 1.0 kg down	1.005	0.983–1.027	0.643			

OR, odds ratio; CI, confidence interval; SMI, skeletal muscle mass index.

**Table 3:** Laboratory associated with covert hepatic encephalopathy.

		Univariate			Multivariate		
Variable	Category	OR	95% CI	*p* Value	OR	95% CI	*p* Value
Platelet count	by 1.0 × 10^3^/μL down	1.003	1.000–1.006	0.198			
Serum albumin	by 0.1 g/dL down	1.056	1.021–1.093	<0.01	1.047	1.011–1.085	<0.01
Total bilirubin	by 0.1 mg/dL up	1.037	1.013–1.062	<0.01			
Prothrombin time	by 1.0% down	1.011	1.001–1.021	0.027			
BUN	by 1.0 mg/dL up	0.993	0.962–1.025	0.667			
Creatinine	by 0.1 mg/dL up	1.006	0.973–1.039	0.730			
Sodium	by 1.0 meq/L down	1.018	0.949–1.091	0.621			
Blood ammonia	by 10 µg/dL up	1.152	1.079–1.229	<0.0001			
Serum 25(OH)D_3_	by 1.0 ng/mL down	1.041	1.011–1.071	<0.01	1.032	1.002–1.063	<0.05
Zinc	by 1.0 µg/dL down	1.012	0.999–1.025	0.052			

OR, odds ratio; CI, confidence interval.

## Data Availability

The original contributions presented in the study are included in the article, further inquiries can be directed to the corresponding author.

## Supplementary Materials

The following supporting information can be downloaded at: https://www.mdpi.com/article/10.3390/jcm14061858/s1. Supplementary Figure S1. Schematic illustration of the study design. Supplementary Figure S2. Receiver operator characteristic curve analysis of the predictive value of serum albumin for covering hepatic encephalopathy in patients under 75 years. (a) The optimal serum albumin cut-off value for predicting cover hepatic encephalopathy was 3.7 g/dL. AUC, area under the curve. (b) Prevalence of cover hepatic encephalopathy according to the cut-off serum albumin value. Supplementary Figure S3. Receiver operator characteristic curve analysis of the predictive value of serum 25(OH)D3 for covering hepatic encephalopathy in patients under 75 years. (a) The optimal serum 25(OH)D3 cut-off value for predicting cover hepatic encephalopathy was 16.4 ng/mL. AUC, area under the curve. (b) Prevalence of cover hepatic encephalopathy according to the cut-off serum 25(OH)D3 value. Supplementary Figure S4. Prevalence of patients with covering hepatic encephalopathy with or without esophageal varices in patients under 75 years. Supplementary Figure S5. Prevalence of covert hepatic encephalopathy according to combined risk factors, including serum albumin, 25(OH)D3, and esophageal varices. Supplementary Table S1. Factors associated with covert hepatic encephalopathy among patients under 75 years. Supplementary Table S2. Laboratory factors associated with covert hepatic encephalopathy among patients under 75 years.
