# Supplementary material for: Association Between Laboratory Values and Covert Hepatic Encephalopathy in Patients with Liver Cirrhosis: A Multicenter, Retrospective Study
Source: J Clin Med. 2025 Mar 10;14(6):1858. doi: 10.3390/jcm14061858 (PMC11942637; doi:10.3390/jcm14061858)
Supplement: Supplementary file 1 [file jcm-14-01858-s001.zip › R1. CHE Table S2.docx]

|  | | **univariate** | | | **multivariate** | | |
| --- | --- | --- | --- | --- | --- | --- | --- |
| **variable** | **category** | **OR** | **95% CI** | **p value** | **OR** | **95% CI** | **p value** |
| Platelet count | by 1.0×10^3^/μL down | 1.003 | 1.000–1.007 | 0.058 |  |  |  |
| Serum albumin | by 0.1 g/dL down | 1.078 | 1.036–1.121 | <0.001 | 1.066 | 1.024–1.111 | <0.01 |
| Total bilirubin | by 0.1 mg/dL up | 1.493 | 1.131–1.972 | <0.01 |  |  |  |
| Prothrombin time | by 1.0 % down | 1.013 | 1.001–1.024 | 0.027 |  |  |  |
| BUN | by 1.0 mg/dL up | 1.005 | 0.965–1.047 | 0.801 |  |  |  |
| Creatinine | by 0.1 mg/dL up | 1.133 | 0.668–1.923 | 0.644 |  |  |  |
| Sodium | by 1.0 meq/L down | 1.022 | 0.941–1.110 | 0.608 |  |  |  |
| Blood ammonia | by 10 µg/dL up | 1.143 | 1.061–1.231 | <0.001 |  |  |  |
| Serum 25(OH)D_3_ | by 1.0 ng/mL down | 1.053 | 1.017–1.090 | <0.01 | 1.039 | 1.003–1.076 | <0.05 |
| Zinc | by 1.0 µg/dL down | 1.023 | 1.008–1.039 | <0.01 |  |  |  |

Supplementary Table 2. Laboratory factors associated with covert hepatic encephalopathy among the patients under 75 years.

OR, odds ratio; CI, confidence interval.
