# Supplementary figures and images for: Association Between Laboratory Values and Covert Hepatic Encephalopathy in Patients with Liver Cirrhosis: A Multicenter, Retrospective Study
Source: J Clin Med. 2025 Mar 10;14(6):1858. doi: 10.3390/jcm14061858 (PMC11942637; doi:10.3390/jcm14061858)

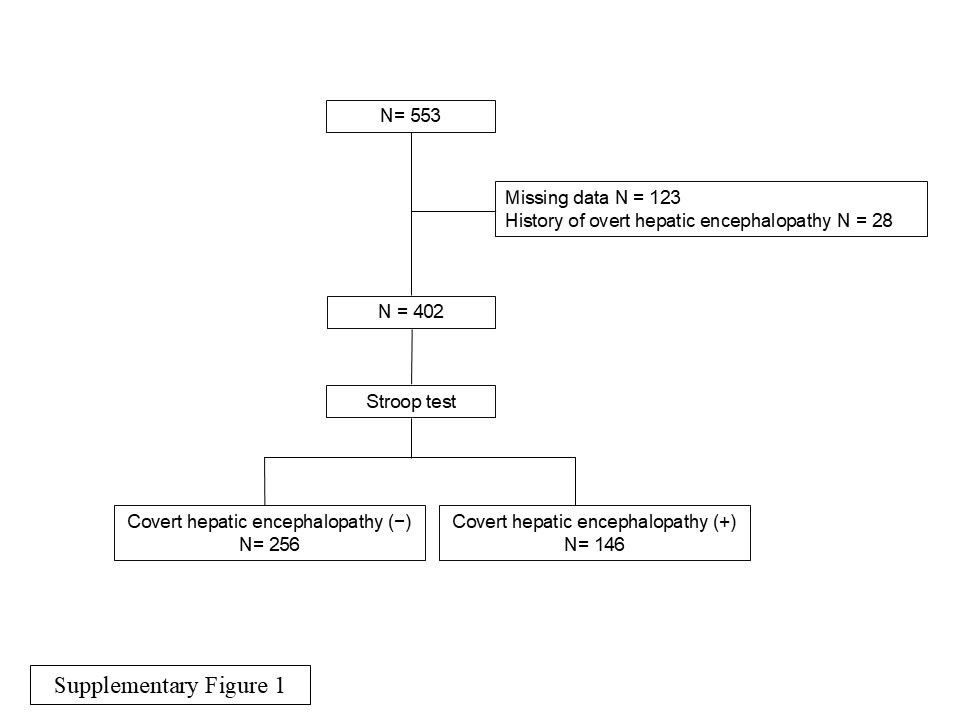

Supplement: Supplementary file 1 [file jcm-14-01858-s001.zip › R1. Figure S1.jpg]

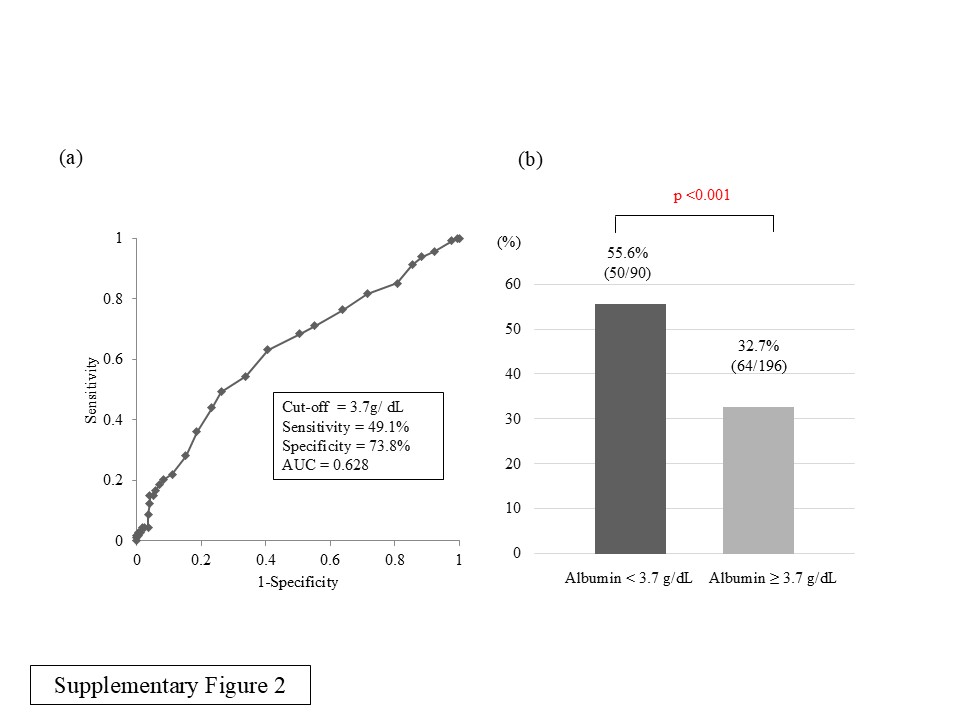

Supplement: Supplementary file 1 [file jcm-14-01858-s001.zip › R1. Figure S2.jpg]

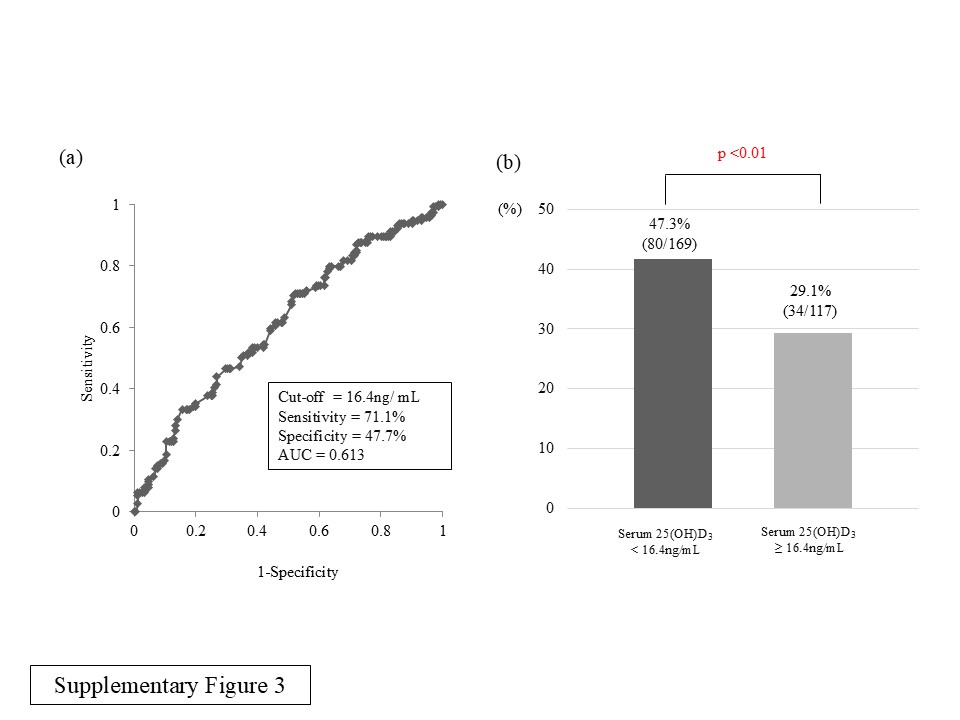

Supplement: Supplementary file 1 [file jcm-14-01858-s001.zip › R1. Figure S3.jpg]

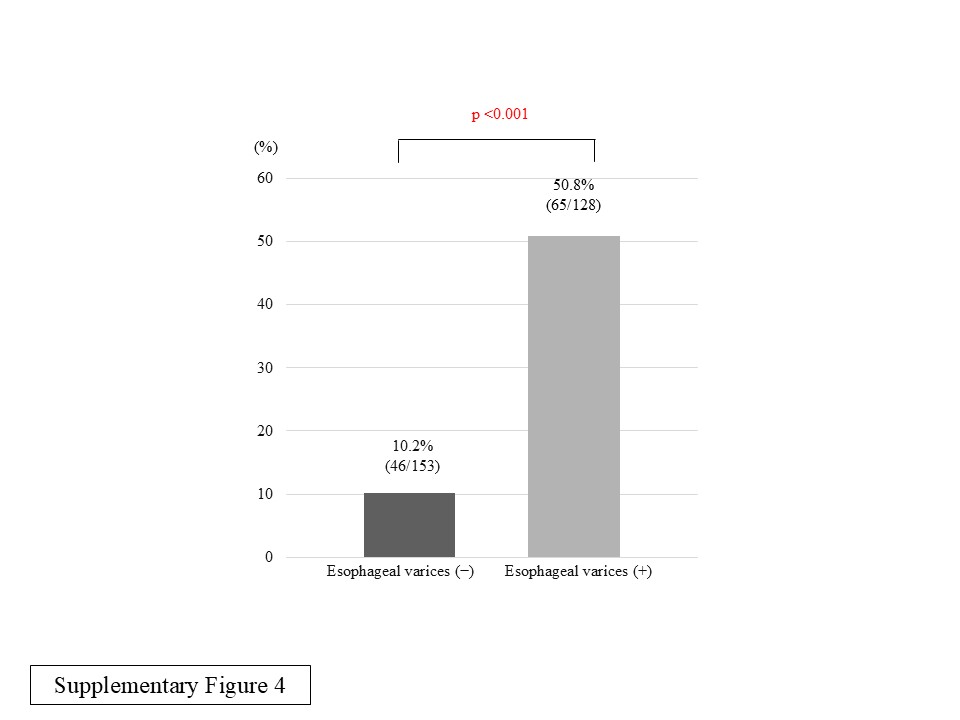

Supplement: Supplementary file 1 [file jcm-14-01858-s001.zip › R1. Figure S4.jpg]

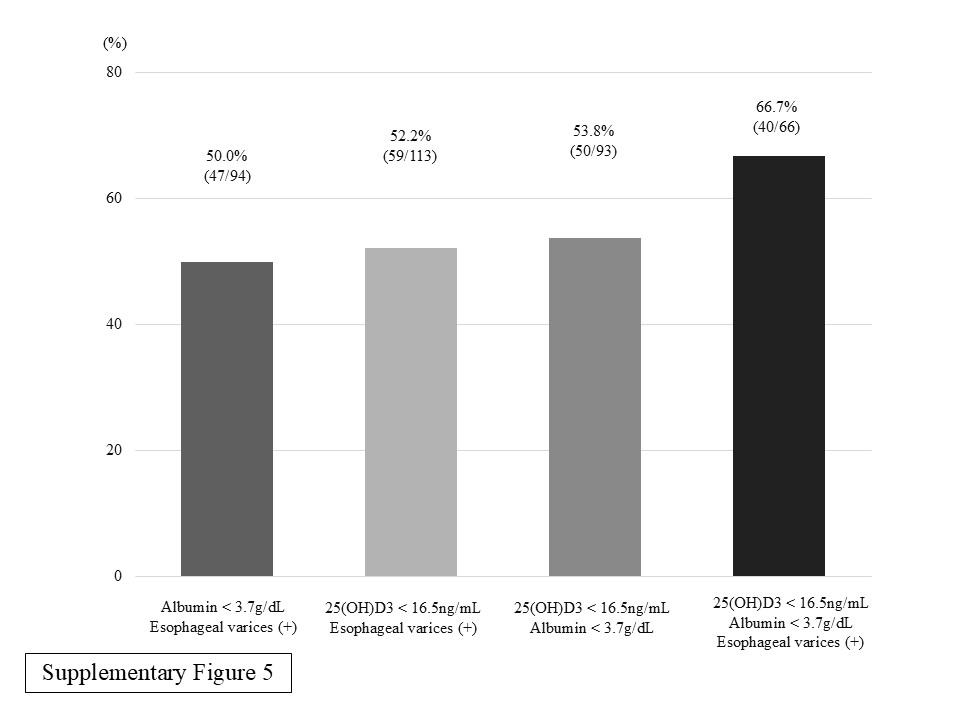

Supplement: Supplementary file 1 [file jcm-14-01858-s001.zip › R1. Figure S5.jpg]
